# Mapping QTLs for Reproductive Stage Salinity Tolerance in Rice Using a Cross between Hasawi and BRRI dhan28

**DOI:** 10.3390/ijms231911376

**Published:** 2022-09-27

**Authors:** Sejuti Mondal, Endang M. Septiningsih, Rakesh K. Singh, Michael J. Thomson

**Affiliations:** 1 Department of Soil and Crop Sciences, Texas A&M University, College Station, TX 77843, USA; 2 Department of Agricultural Sciences, Texas State University, San Marcos, TX 78666, USA; 3 International Center for Biosaline Agriculture, Dubai 14660, United Arab Emirates

**Keywords:** rice (*Oryza sativa* L.), salinity stress, quantitative trait locus (QTL), single nucleotide polymorphism (SNP) markers, skim sequencing

## Abstract

Salinity stress is a major constraint to rice production in many coastal regions due to saline groundwater and river sources, especially during the dry season in coastal areas when seawater intrudes further inland due to reduced river flows. Since salinity tolerance is a complex trait, breeding efforts can be assisted by mapping quantitative trait loci (QTLs) for complementary salt tolerance mechanisms, which can then be combined to provide higher levels of tolerance. While an abundance of seedling stage salinity tolerance QTLs have been mapped, few studies have investigated reproductive stage tolerance in rice due to the difficulty of achieving reliable stage-specific phenotyping techniques. In the current study, a BC_1_F_2_ mapping population consisting of 435 individuals derived from a cross between a salt-tolerant Saudi Arabian variety, Hasawi, and a salt-sensitive Bangladeshi variety, BRRI dhan28, was evaluated for yield components after exposure to EC 10 dS/m salinity stress during the reproductive stage. After selecting tolerant and sensitive progeny, 190 individuals were genotyped by skim sequencing, resulting in 6209 high quality single nucleotide polymorphic (SNP) markers. Subsequently, a total of 40 QTLs were identified, of which 24 were for key traits, including productive tillers, number and percent filled spikelets, and grain yield under stress. Importantly, three yield-related QTLs, one each for productive tillers (*qPT3.1*), number of filled spikelets (*qNFS3.1*) and grain yield (*qGY3.1*) under salinity stress, were mapped at the same position (6.7 Mb or 26.1 cM) on chromosome 3, which had not previously been associated with grain yield under salinity stress. These QTLs can be investigated further to dissect the molecular mechanisms underlying reproductive stage salinity tolerance in rice.

## 1. Introduction

Rice (*Oryza sativa* L.) is the major staple for almost half of the world’s population. Sustained rice production is essential for food security of many rice-consuming countries across the globe. Unfavorable environmental conditions such as salinity, drought, heat, and submergence pose a huge threat to rice productivity and challenge future food security. Among all the abiotic stresses, salinity is the second most prevalent problem affecting rice productivity worldwide [1] and poses a serious threat to rice-based farming systems, especially in precarious coastal zones. Aside from coastal areas, about 20% of irrigated and 8% of rainfed agricultural land [2] and about one-third of the irrigated rice growing areas are affected by salinity [3]. The salt-affected areas are expected to increase due to the adverse effect of climate change and sea level rise [4]. Therefore, improving the productivity of crops, especially rice as a major staple, in salt-affected areas of the world is considered essential to meet the increasing food demand and sustained food security.

Salinity tolerance is a complex trait and is recognized by multiple mechanisms. Salt injury can be reduced by limiting the amount of Na^+^ in the cytoplasm, which depends on the mechanisms of Na^+^ extrusion from roots, unloading Na^+^ from the xylem, and sequestration of Na^+^ into vacuoles [5]. In addition to Na+ exclusion and sequestration, the presence of cytosolic K^+^, osmolytes, and compatible solutes also play an important role in salinity tolerance by maintaining ion homeostasis. Moreover, compartmentalization of Na^+^ into older leaves and regulation of reactive oxygen species (ROS) can also play a role in salt tolerance in rice [5]. Understanding the underlying genetic mechanisms will help breeders develop strategies to combine these traits into rice genotypes to provide higher levels of salinity tolerance.

Salinity affects plant growth during all developmental stages. Rice is relatively tolerant to salinity stress during germination, tillering and maturity; but is very sensitive at the early seedling and reproductive stages [6,7]. The reproductive stage is crucial as it directly translates into grain yield. Although salinity at the reproductive stage depresses grain yield much more than in the vegetative stage, there are few studies on rice for salinity tolerance at the reproductive stage due to the difficulty of achieving reliable stage-specific phenotyping techniques [8]. Recent advances, however, have now enabled more effective genetic studies of reproductive stage tolerance in rice, allowing for comparisons across genetic donors and the genetic control of tolerance between the seeding and reproductive stages [9].

Progress in breeding salt-tolerant varieties is challenging due to genetic complexity and interaction with environmental factors that affect the phenotype. As a polygenic trait, the genetics behind salinity tolerance can be revealed using quantitative trait locus (QTL) analysis [10,11]. Advances in molecular marker technology have enabled the dissection of the molecular mechanisms of salinity tolerance to identify major-effect QTLs [12,13,14,15,16,17,18,19]. More recently, single nucleotide polymorphisms (SNPs) have become the marker of choice for high throughput genotyping applications because they are ubiquitous in eukaryotic genomes, cost-effective to assay using automated platforms, and biallelic in nature, which is useful for allele calling and data analysis [20]. 

Identifying beneficial alleles and introgressing those alleles into a popular variety is a successful breeding approach for sustaining productivity in stress-prone areas. Of the five subgroups of cultivated Asian rice (*Oryza sativa*), most studies have investigated salt-tolerant *indica* accessions, such as Pokkali or Nona Bokra. The *aus* landrace “Hasawi”, originating from Saudi Arabia, however, has been found to have high Na+ exclusion and early seedling vigor [21,22,23]. It is a highly salt-tolerant genotype that is well adapted to soil salinity and drought. However, it has some undesirable characteristics, including tall plant height leading to lodging, late flowering, and photoperiod sensitivity [23]. A previous report that crossed Hasawi with three African varieties detected several grain yield-enhancing QTLs from Hasawi at reproductive stage under salinity stress, suggesting that Hasawi is a promising genetic donor for further study [23]. On the other hand, BRRI dhan28, an *indica* cultivar, is a Bangladeshi mega variety that has all the desirable characteristics for higher productivity except that it is sensitive to salinity. In the current study, a BC_1_F_2_ mapping population developed from the cross between Hasawi and BRRI dhan28 was used to map quantitative trait loci (QTLs) associated with salinity-related traits using a high-density SNP linkage map. Thus, the objective of this study is to identify QTLs for salinity tolerance from the *aus* variety Hasawi that have the potential to improve the salt-sensitive *indica* variety BRRI dhan28 and provide genetic materials to further investigate the salinity tolerance mechanisms of *aus*-derived alleles in an *indica* genetic background. 

## 2. Results

### 2.1. Responses of the Parental Lines and BC1F2 Progenies to Salt Stress

**Figure 1:**
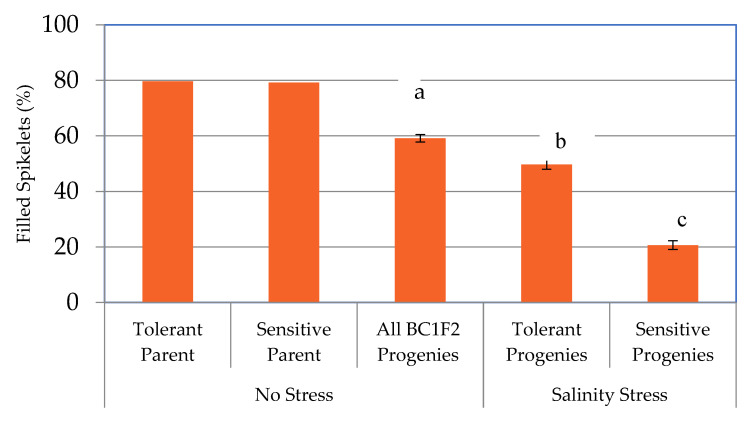
Percent filled spikelets per plant of tolerant and sensitive parents under no stress and their BC_1_F_2_ progenies under no stress and salt stress condition. Vertical and capped bar indicates standard error of the mean percent filled spikelets of 153 progenies under no-stress, 78 tolerant and 112 sensitive progenies under salinity stress. Values with the same letter are not significantly different at 5% level of significance.

A population of 435 BC_1_F_2_ individuals from a cross between Hasawi × BRRI dhan28 was grown under salt stress (EC 10 dS/m) during the reproductive stage. A wide range of phenotypic variability was observed across the population for plant height, number of productive tillers, number and percentage of filled spikelets and grain yield under the stress conditions (Supplementary Table S1). To implement a selective genotyping approach, 78 of the most tolerant and 112 of the most sensitive BC_1_F_2_ progenies were selected based on grain yield and the standard evaluation system (SES) score visual symptoms. After undergoing salinity stress during the reproductive stage, significant differences were observed between tolerant and sensitive progenies in plant height, productive tillers, number and percentage of filled spikelets, number of unfilled spikelets, grain yield and sodium-potassium (Na^+^-K^+^) ratio (Supplementary Table S2). The average reduction of trait values for sensitive genotypes compared to tolerant genotypes was 15.5% for plant height, 40.1% for productive tillers, 83.2% for the number of filled spikelets, 58.4% for the percentage of filled spikelets, and 87.1% for grain yield per plant (Supplementary Table S1). Although the number of filled spikelets varied significantly between tolerant and sensitive individuals, it also varied significantly (*p* < 0.001) between stress and control (no-stress) treatments (Supplementary Figure S1 and Supplementary Table S2). Interestingly, no significant variation in the number of filled spikelets per plant was found between tolerant progenies under stress (735.52) and control progenies (637.48). However, in the case of percent filled spikelets, the control and tolerant progenies varied significantly (Figure 1 and Supplementary Table S2). On the other hand, the number of unfilled spikelets per plant also differed significantly (*p* < 0.001) between no-stress and salinity treatments as well as between tolerant and control treatments (Supplementary Table S2).

The grain yield of the progenies varied from 2.5 g/plant to 80.4 g/plant under control conditions and from 0.0 to 43.5 g/plant under salinity stress (Supplementary Figure S2 and Supplementary Table S1). It differed significantly (*p* < 0.01) between control and salinity stress treatments (Supplementary Figure S2 and Supplementary Table S2). The mean grain yield of the tolerant progenies of the Hasawi × BRRI dhan28 population was 15.1 g/plant and the sensitive progenies was 2.3 g/plant, while the control plants had a mean of 16.5 g/plant (Supplementary Table S1 and Supplementary Figure S2). As with the number of filled spikelets, the tolerant progenies of Hasawi × BRRI dhan28 did not significantly vary with the no-stress treatment (Supplementary Table S2 and Supplementary Figure S2). Overall, the yield reduction of the tolerant progenies over those grown under control conditions was 8.6% (Supplementary Table S1 and Supplementary Figure S2). Moreover, the Na^+^-K^+^ ratio significantly varied between tolerant and sensitive progenies, and the concentration of K^+^ was higher than that of Na^+^ in the tolerant progenies (Supplementary Table S2).

### 2.2. Correlation Analysis of Yield and Agronomic Components

**Figure 2:**
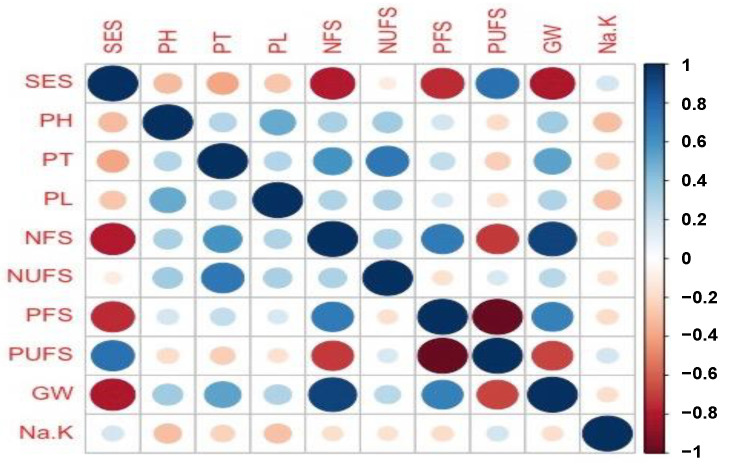
Correlation coefficients for grain yield and agronomic components of BC_1_F_2_ individuals from Hasawi × BRRI dhan28 under salinity stress of 10 dS/m at the reproductive stage of rice plant.

Correlation analysis among the salinity scoring using SES, plant height (PH), productive tillers per plant (PT), panicle length (PL), number of filled spikelets per plant (NFS), number of unfilled spikelets per plant (NUFS), percent filled spikelets (PFS), percent unfilled spikelets (PUFS), grain weight/yield (GW) and sodium-potassium ratio (Na^+^-K^+^) were performed for 435 BC_1_F_2_ progenies (Figure 2). Highly significant positive correlations between grain yield and the number of filled spikelets (r = 0.93), percent filled spikelet (r = 0.68), and productive tillers (r = 0.54) were observed across the 435 BC_1_F_2_ individuals. The percent unfilled spikelets (r = −0.68) and SES score (r = −0.80) also showed a significant but negative correlation with the grain yield. Other than grain yield, salinity evaluation score showed negative and highly significant correlation with plant height, productive tiller per plant, panicle length, number of filled spikelets per plant and percent filled spikelets (Figure 2).

### 2.3. Trait Distributions

**Figure 3:**
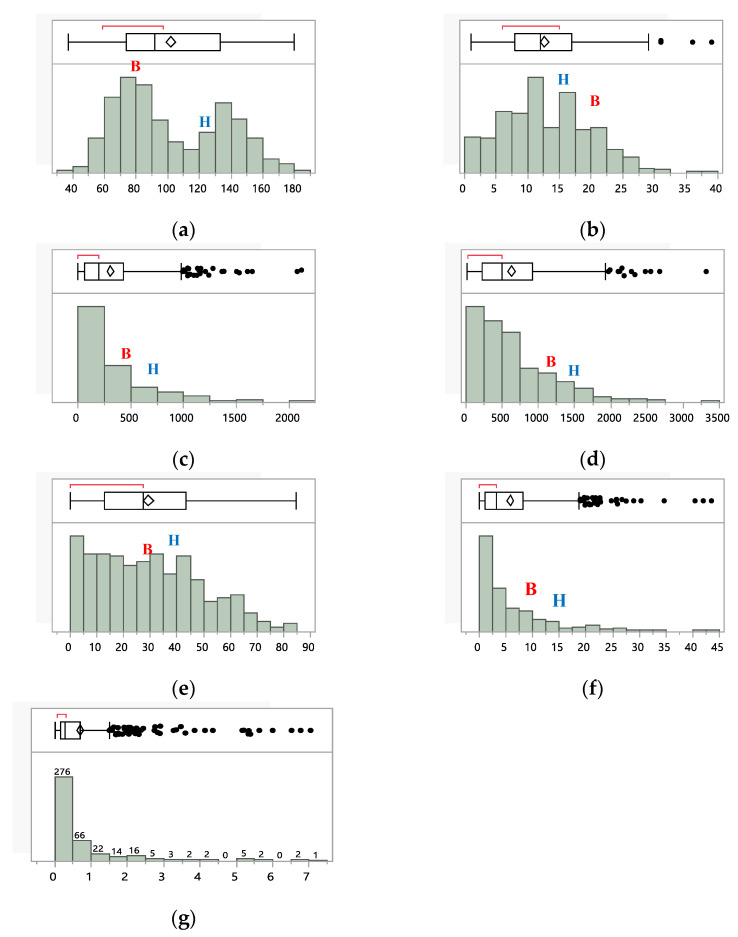
Trait frequency distribution of the agronomic traits and Na^+^-K^+^ ratio of the 435 BC_1_F_2_ progenies grown under salinity stress of EC 10 dS/m at the reproductive stage. The parental means are also shown: BRRI dhan28 (B) and Hasawi (H).(**a**) Plant Height (cm). (**b**) Productive Tiller (no/plant). (**c**) Filled Spikelets (no/plant). (**d**) Unfilled Spikelets (no/plant). (**e**) Filled Spikelets (%). (**f**) Grain Yield (g/plant). (**g**) Na^+^-K^+^ Ratio.

Out of the 435 BC_1_F_2_ progenies of Hasawi × BRRI dhan28, 124 plants were categorized as tolerant to moderately tolerant, and 311 plants were categorized as sensitive to highly sensitive. Trait frequency distributions of BC_1_F_2_ progenies and the parents showed a wide range of variation (Figure 3). Phenotypic variation of the plant height was positively skewed, and 95.2% ranged from 50 cm to 160 cm. Out of 435 BC_1_F_2_ progenies, 33.1% were taller than the tolerant parent, Hasawi, and 23.9% had shorter plants than the sensitive parent, BRRI dhan28. Overall, 43% of the total population had plant height between the tolerant and sensitive plants. Similarly, the phenotypic variation was positively skewed for the number of productive tillers, and 43.2% of the total population had more productive tillers than the tolerant parent, while 17.0% had more productive tillers than the sensitive parent, suggesting transgressive segregation in both directions. Among all the BC_1_F_2_ population, 17.9% individuals had a greater number of filled spikelets per plant than the tolerant parent, and 69% had less number of filled spikelets per plant than the sensitive parent (Figure 3). Out of 435 BC_1_F_2_ progenies, 83.7% of individuals had percent filled spikelets ranging from 0 to 50%. Among them, 45.3% (197 individuals) had a greater percentage of filled spikelets than the tolerant parent, and 38.4% had a smaller percentage of filled spikelets than the sensitive parent. Overall, 16.3% (71 BC_1_F_2_ individuals) of the individuals had the filled spikelets between tolerant and sensitive parents (Figure 3). In grain yield, the mean of the whole population for the Hasawi × BRRI dhan28 was 5.85 g/plant, and that varies from 0 to 43.5 g/plant (Supplementary Table S1). Among them, 78.9% of individuals had grain yield ranging from 0 to 10 g/plant and 11.7% of individuals showed higher grain yield than the tolerant parent. On the other hand, 68.3% had lower grain yield than the sensitive parent (Figure 3). This distribution is highly positively skewed. The distribution of this population for Na^+^-K^+^ ratio is positively skewed (+4.458), and among the progenies, the 82.2% individuals ranged from 0 to 1.

### 2.4. QTL Mapping for Salinity Tolerance at Reproductive Stage

**Figure 4:**
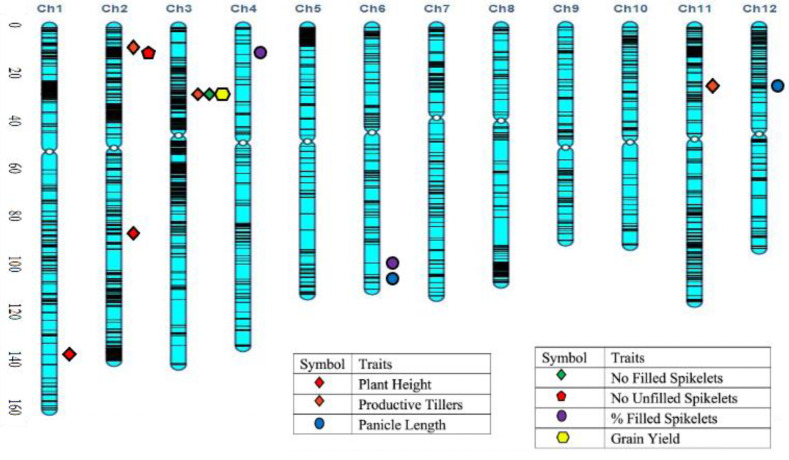
Genetic linkage map using 6209 SNP markers based on the BC_1_F_2_ mapping population of Hasawi × BRRI dhan28 showing QTLs mapped under salinity stress of EC 10 dS/m at the reproductive stage of rice. Significant QTLs are shown at the right side of each chromosome (Ch) based on the genetic position in centimorgan (cM) of the SNP markers.

Based on the phenotypic data under salt stress, two extreme tails of the trait distributions for grain yield and SES scores were used to select 78 highly tolerant plants and 112 highly sensitive plants for a selective genotyping strategy. These 190 BC_1_F_2_ individuals plus the two parents were genotyped with 6209 polymorphic SNP markers using a skim sequencing approach. QTL mapping was performed using the phenotypic data collected for plant height (PH), productive tillers (PT), number of filled spikelets (NFS), number of unfilled spikelets (NUFS), percent filled spikelet (PFS), percent unfilled spikelets (PUFS), grain yield (GY) and Na^+^-K^+^ ratio. Using a LOD 3.0 threshold, a total of 40 QTLs were identified across the seven traits on eleven chromosomes, except for chromosome 10, with phenotypic variation ranging from 1.66% to 27.03% (Figure 4 and Table 1).

**Figure 5:**
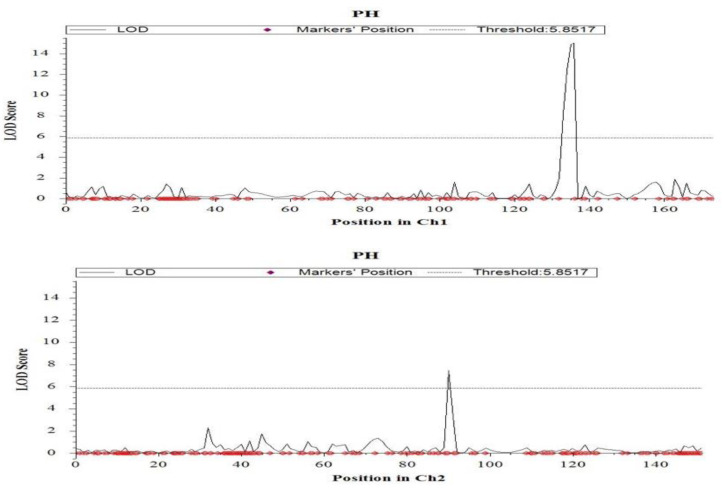
Chromosome locations of QTLs for plant height under salinity stress based on a significant threshold of LOD 3.0 using ICIMapping. The horizontal line indicates the significant LOD threshold at 95% confidence levels based on 1000 permutations.

**Figure 6:**
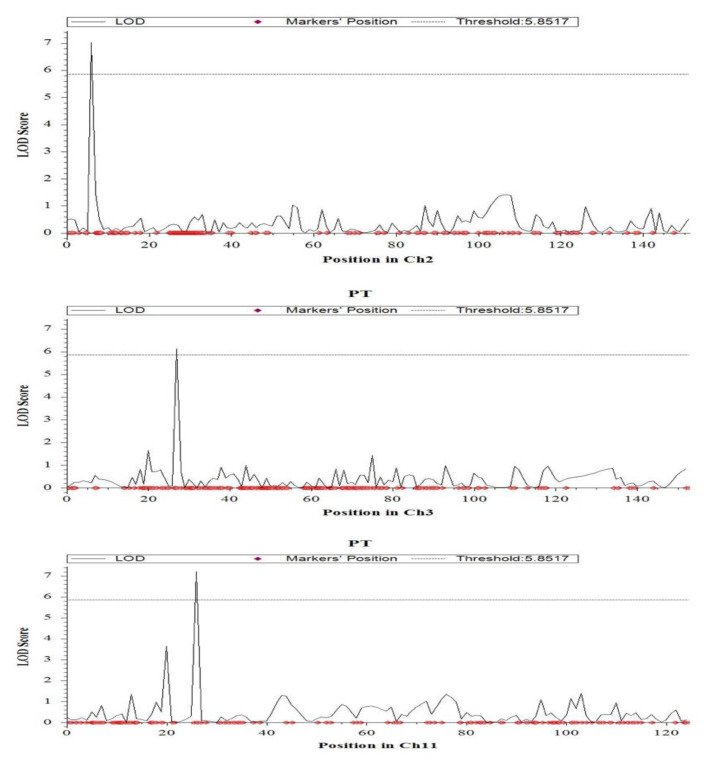
Chromosome locations of QTLs for productive tillers per plant under salinity stress based on a significance threshold of LOD 3.0 using ICIMapping on chromosomes 2, 3, and 11. The horizontal line indicates the significant LOD threshold at 95% confidence levels based on 1000 permutations.

For plant height, two significant (*p* < 0.05) QTLs were identified on chromosome 1 (*qPH1.1*) and on chromosome 2 (*qPH2.1*). They were mapped at 139.7 cM, and 91.2 cM, with the marker intervals of CM020682.1_33842918_1–CM020682.1_34965920_1 and CM020683.1_22803914_2–CM020683.1_23139808_2, respectively (Figure 5 and Table 1). The largest effect was found at *qPH1.1* with a LOD value of 14.9 which can explain 27% phenotypic variation, followed by *qPH2.1* with the LOD of 7.5 which explains 9.6% of the phenotypic variation. Both the QTLs were contributed by the tolerant parent, Hasawi. Three significant (*p* < 0.05) QTLs were identified for productive tillers on the short arm of chromosome 2 (*qPT2.1*), chromosome 3 (*qPT3.1*) and chromosome 11 (*qPT11.1)* (Table 1 and Figure 6). They were mapped at 7.2 cM, 27.1 cM, and 26.4 cM. Of the significant QTLs, the largest effect was found at *qPT11.1* with a LOD value of 7.2 which can explain 13.4% of the phenotypic variation (Table 1 and Figure 6). The additive effect of *qPT11.1* is positive, indicating that the sensitive parent, BRRI dhan28 is responsible for this QTL. The second largest effect was observed in *qPT2.1* with a LOD of 7.0, followed by *qPT3.1* with a LOD value of 6.1. Both the QTLs can explain about 12% phenotypic variation and had a negative additive effect that indicated the tolerant parent (Hasawi) was responsible for these QTLs.

**Figure 7:**
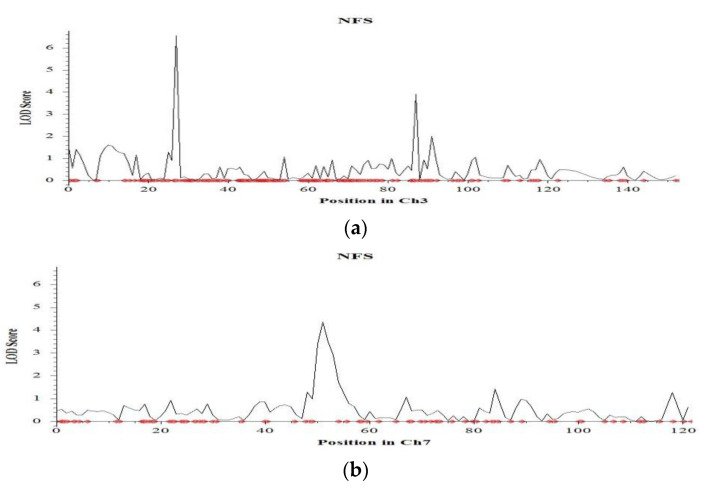

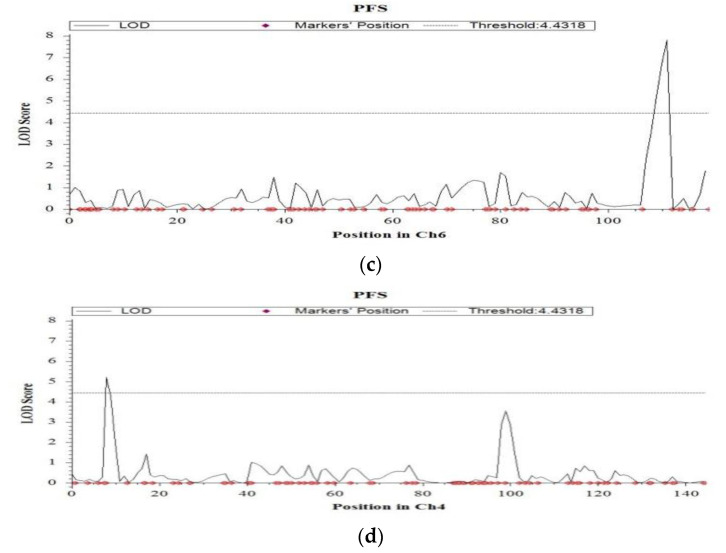
Chromosome locations of QTLs for number and percent filled spikelets under salinity stress based on a significant threshold of LOD 3.0 using ICIMapping on: (**a**) chromosome 3, (**b**) chromosome 7, (**c**) chromosome 6, and (**d**) chromosome 4. The horizontal line indicates the significant a LOD threshold at 95% confidence levels based on 1000 permutations.

Seven QTLs were identified for the number of filled spikelets per plant on chromosomes 3, 6, 7, 8, 11 and 12 (Table 1). Among the QTLs, the largest effect was found in *qNFS3.1* with a LOD value of 6.6 which can explain 17.9% phenotypic variation, followed by qNFS7.1 with a LOD value of 3.5 which can explain 17.4% phenotypic variation (Table 1 and Figure 7a,b). The QTLs were mapped on the short arm of chromosome 3 at 27.1 cM and chromosome 7 at 51.8 cM. In both cases, the additive effect was negative, indicating the tolerant parent (Hasawi) is responsible for both the QTLs. Similarly, Hasawi was also responsible for contributing the allele to the QTL for the number of unfilled spikelets (Table 1). Only one QTL was identified for this trait on the short arm of chromosome 2 with a LOD value of 5.2 and can explain 11.4% phenotypic variation. 

In the case of percent filled spikelets; two QTLs (*qPFS4.1* and *qPFS6.1*) were significant (*p* < 0.05) based on 1000 permutations and were mapped on the short arm of chromosome 4 at 9.4 cM and the long arm of the chromosome 6 and at 111.9 cM with a LOD value of 4.3 and 7.8 that explains about 15% phenotypic variations, respectively (Table 1 and Figure 7c,d). The QTL *qPFS4.1* was mapped with the marker interval of CM020685.1_2019282_4–CM020685.1_3262402_4 and *qPFS6.1* was mapped in between CM020687.1_26815088_6–CM020687.1_28217292_6. The percent-filled spikelets exhibited a positive additive effect for both the QTLs, indicating that the sensitive parent, BRRI dhan28 is responsible for percent-filled spikelets.

**Figure 8:**
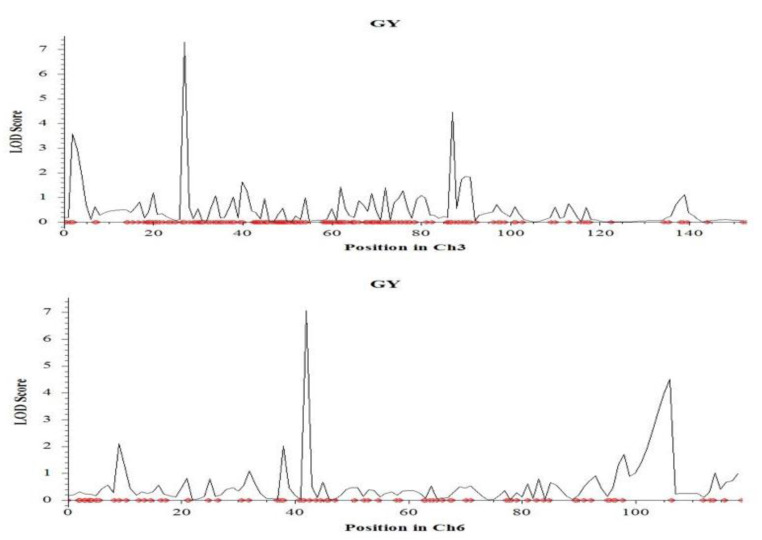
Chromosome locations of QTLs for grain yield per plant under salinity stress based on a significant threshold of LOD 3.0 using ICIMapping on: chromosome 3 and chromosome 6. The horizontal line indicates the significant LOD threshold at 95% confidence levels based on 1000 permutations.

Grain yield is the ultimate stress indicator, and eight QTLs were identified for this trait on chromosomes 1, 3, 4, 6 and 9 under salinity stress at the reproductive stage of rice (Table 1). Among them, the largest effect was found in *qGY3.1* mapped on the short arm of chromosome 3 with a LOD value of 7.3 that can explain 11.6% phenotypic variation followed by *qGY6.1* on chromosome 6 (mapped on the short arm) with a LOD value of 7.1 that can explain 19.2% phenotypic variation (Figure 8). *qGY3.1* was located in between the marker CM020684.1_6731611_3 and CM020684.1_6810365_3, and the additive effect was negative, indicating that the tolerant parent (Hasawi) contributed alleles for this QTL.

For Na^+^-K^+^ ratio, twelve QTLs were identified on chromosomes 1, 3, 4, 5, 6, 7, 9, 11 and 12 under salinity stress at the reproductive stage of rice (Table 1). Among them, the largest effect was found in *qNaK3.2* mapped on the long arm of chromosome 3 with a LOD value of 28.8 that can explain 3.0% phenotypic variation. Interestingly, the additive effect was positive, indicating that the sensitive parent (BRRI dhan28) contributed alleles for this QTL. Although the LOD value is much higher for rest of the QTLs, the phenotypic variations of the identified QTLs were less than 2%. 

## 3. Discussion

In this study, a population of 588 BC_1_F_2_ individuals derived from the cross between a salt-sensitive variety, BRRI dhan28, and a salt-tolerant variety, Hasawi, were evaluated under salinity stress of EC 10 dS/m to identify quantitative trait loci (QTLs) for salinity tolerance at the reproductive stage of rice. Continuous 20 days of salt stress was applied at the booting stage to 435 BC_1_F_2_ progenies, and the remaining 153 progenies were grown under non-stress (control) conditions. Among 435 progenies, 28.5% were classified as tolerant, and the rest as sensitive based on their SES score and grain yield. A selective mapping approach was successfully implemented by genotyping 78 extremely tolerant and 112 highly sensitive progenies for QTL mapping using 6209 SNP markers from skim sequencing. The high-density map allowed for a comprehensive view of the genome for QTL mapping. Almost all the traits differed significantly under salinity stress over those obtained under non-stress conditions, except the percent filled grains. Likewise, all traits of the tolerant and sensitive progenies under salt stress differed significantly as well. Yield reduction between tolerant progenies of Hasawi × BRRI dhan28 grown under salinity stress and control condition was only 8.6%. The correlation analysis indicated a positive and significant (*p* < 0.001) correlation between grain yield and the number of filled spikelets, percent filled spikelets, and productive tillers and a significantly negative (*p* < 0.001) correlation with SES score and percent unfilled spikelets of the progenies.

Productive tillers, number of filled spikelets and grain yield are the most important traits of salinity tolerance at the reproductive stage in rice. Additionally, SES score and grain yield are negatively correlated; therefore, SES score might be an initial stress indicator to identify salt-tolerant and salt-sensitive genotypes. In addition to SES score, the final grain yield, filled spikelets and productive tillers are considered the primary stress indicators for salinity tolerance in rice. In this study, QTLs related to salinity tolerance were mapped using a novel population of Hasawi × BRRI dhan28. Many of the identified QTLs co-located with previously reported QTLs, confirming the importance of these loci, while others are potentially novel, as described below.

A total of 40 QTLs were identified for plant height (PH), productive tillers (PT), panicle length (PL), number of filled spikelets (NFS), number of unfilled spikelets (NUFS), percent filled spikelets (PFS), grain yield (GY) and Na^+^-K^+^ ratio. In this study, *qPH1.1* and *qPH2.1* were mapped on the long arm of chromosomes 1 and 2 at 139.67 cM and 91.23 cM, respectively. QTL, *qPH2.1* lies within 100 kbp (4 cM) of a previously reported dwarf QTL [24]. Likewise, *qPH1.1* was also mapped near a previously reported locus, in this case, the semi-dwarf 1 (*sd1)* gene. The *sd1* gene is a major gene controlling plant height in rice that significantly increased rice yield throughout Asia during the Green Revolution in the 1960s and onward by reducing lodging for heavily fertilized rice. The phenotype of this gene is dwarfism which was the result of the deficiency of the plant growth hormone GA in the elongating stem. The location of this gene was on the long arm chromosome 1 and was mapped at 149.1 cM, which is close to the location of *qPH1.1* in the current study [25]. Hasawi is a tall traditional rice variety, while BRRI dhan28 is semi-dwarf, which validates that the current QTL study has been implemented correctly as the Hasawi allele contributed to plant height at *qPH1.1*.

The number of productive tillers per plant is an important trait for determining the salinity tolerance in rice at reproductive stage. No previously reported QTLs for productive tiller number under salt stress were found on chromosomes 2, 3 and 11 in the regions of the currently mapped QTLs. So, these QTLs could be considered novel. The number of filled spikelets per plant is one of the most important traits for grain yield, especially salinity tolerance, particularly at the reproductive stage in rice. In this study, seven QTLs for this trait were detected on chromosomes 3, 6, 7, 8, 11 and 12. Among them the largest effect of the QTL, *qNFS3.1* was found on chromosome 3 at 27.08 cM. Previously mapped QTLs for the number of filled spikelets per plant under salt stress have been identified on chromosome 2 [26], and four QTLs have been detected on chromosomes 2, 4, 6 and 10 for this trait [27] and one major QTL qSSISFH8.1 for spikelet fertility was identified and fine mapped for salinity tolerance at the reproductive stage on chromosome 8 in CSR27 [28]. So far, no QTL for this trait has been found on chromosome 3; therefore, *qNFS3.1* identified in this study can be considered a novel QTL.

Generally, due to salinity stress, the percentage of filled spikelets will be reduced. However, when the percent filled spikelets are moderate to high, the population is considered tolerant. In this study, *qPFS4.1* and *qPFS6.1* crossed the threshold level (*p* < 0.05) based on 1000 permutations. A previously reported QTL at 2.71 cM on chromosome 4 (*qSNP-4a*) was found for the same trait with 16.1% phenotypic variation of 16.7 LOD value from the reciprocal introgression line (IL) which is near to qPFS4.1 (9.36 cM) [29]. Another QTL for this trait was identified from an RIL population on chromosome 4 at 4.55 cM, which is near to the identified QTL in the current study [30]. Moreover, additional QTLs for the number of filled spikelets have been found on chromosome 2 but at different positions [24,25].

The number of unfilled spikelets tends to be increased due to salinity stress and is considered an important parameter to know the stress and magnitude of salinity on the performance of a crop. In this study, one QTL was identified on the short arm of chromosome 2 (*qNUFS2.1*) at 10.23 cM. A previously reported pollen-detective mutant, Collapsed Abnormal Pollen 1 (CAP1), was isolated from insertional mutant lines of rice [31] and was located on the short arm chromosome 2 at 8.92 cM, in a similar region as *qNUF2.1*, suggesting that this locus may also be detectable using natural variation. 

Grain yield is the ultimate factor in determining salinity tolerance at the reproductive stage of rice. In the current study, the *qGY1.1* QTL on chromosome 1 had a LOD of 6.2 and was in a similar region as the previously reported QTL for grain yield on chromosome 1 with 8.4% phenotypic variation and 3.7 LOD value using 164 RIL population of Milyang 23/Gihobyeo [32]. In addition, Bimpong et al. [23] identified one QTL on chromosome 1 with LOD value of 4.7 and 12.0% phenotypic variation using F2 population derived from BG90-2 and Hasawi. The *qGY1.1* was found in the long arm and no QTL was found on the short arm of chromosome 1 or near *Saltol* QTL. Similarly, one QTL for Na^+^-K^+^ in this study was identified on the long arm of chromosome 1 and no QTL was found on the short arm of chromosome 1 near the *SKC1* gene [33]. This confirm the hypothesis that genes controlling tolerance at seedling and reproductive stage are very different. Next, the QTL *qGY3.1* on chromosome 3 in this study showed the largest effect with a LOD value of 7.3. One of the most important findings from this study is that three yield-related QTLs, one each for productive tillers (*qPT3.1*), number of filled spikelets (*qNFS3.1*) and grain yield (*qGY3.1*), were all mapped at the same position (6.73 Mb or 26.07 cM) on chromosome 3. Two previous studies also identified grain yield QTLs on chromosome 3 within a 100 kb region of *qGY3.1*, although not under salinity stress [34,35]. Similarly, another QTL was identified for grain yield (Gy3a) from a double haploid population with a LOD value 3.11 that explained 12.1% phenotypic variation and mapped at around 5.5 Mb or 21.9 cM on chromosome 3 [35]; which is very near to the identified QTLs (*qPT3.1*, *qNFS3.1* and *qGY3.1*). However, that yield QTL (Gy3a) was related to rationing ability of rice under non-stress conditions, not related to salinity stress. Therefore, the QTL cluster on chromosome 3 from the current study presents a promising target for further study.

## 4. Materials and Methods

### 4.1. Plant Materials

A salt-tolerant Saudi Arabian variety, Hasawi, and a salt-sensitive Bangladeshi rice variety, BRRI dhan28, both in the *indica* subgroup, were used as the parents to develop a mapping population. Since Hasawi is an unimproved traditional variety, one generation of backcrossing was performed to reduce the proportion of negative traits in the population. The BC_1_F_2_ QTL mapping population consisting of 588 BC_1_F_2_ lines was generated at the International Rice Research Institute (IRRI), Philippines crossing program to generate F_1_ and then backcross to the recurrent parent, BRRI dhan28 to produce the BC_1_F_2_ progenies. Parents for each population were used as the checks for the respective population. These progenies were evaluated in the wet season (July–December 2018) under control conditions (n = 153 progenies) and salt stress of EC 10 dS/m (n = 435 progenies) in a concrete tank at IRRI, Philippines. 

### 4.2. Experimental Design and Set-Up

The phenotypic experiments with EC 10 dS/m salinity level and control treatment were set up at IRRI using a Completely Randomized Design (CRD). A set of 588 young seedlings derived from the cross Hasawi × BRRI dhan28 was sown in pots, and those pots were transferred into three large concrete tanks having a dimension of 680 cm × 105 cm × 22 cm (length × width × depth). The concrete tanks were filled with ordinary tap water until the reproductive stage, when salt (NaCl) was added to EC 10 dS/m. These 588 individuals, 435 in saline treatment and 153 in normal water treatment (control), were used for phenotyping under a natural environment, with the exception that rain shelters were placed at about 1.5 m above the ground to protect the saline treatments from rains at the reproductive stage. 

### 4.3. Phenotypic Evaluation

#### 4.3.1. Seedling Preparation and Management

Twenty-five days old seedlings were transplanted manually in the perforated cylindrical pots containing a plastic sieve bag filled with sterilized soil up to about 1 cm above the topmost circle of the holes, about 3 cm below the top of the cylinder. The sterilized soil was fertilized with 50, 25, and 25 mg of N, P, and K per kilogram of soil, respectively [8].

#### 4.3.2. Salinity Tolerance Assessment at Reproductive Stage

As described by Ahemdizadeh [8], the pots were placed in the concrete tank after transplanting. The concrete tanks were filled with normal tap water for the growth and development of the rice plant. A water depth of 12–14 cm was maintained to keep the bottom 75% of the pots underwater throughout the growing season of rice. All the plants were grown in normal water until the appearance of the first flag leaf. All the leaves except the flag and penultimate leaves were pruned to facilitate the direct movement of salt to the reproductive organs avoiding salt compartmentalization [8]. After pruning, 435 plants were transferred into saline water of EC 10 dS/m for 20 days, and 153 plants were kept under control conditions. After 20 days, the salinity-treated plants were shifted back to the normal water, and the first flag leaf was collected to determine the Na^+^ and K^+^ ion concentration of the leaf samples [8]. 

To analyze the ion concentration, stock solution was diluted 10 times (1 mL of stock solution and 9 mL of nanopore water) and Perkin-Elmer Analyst 300 atomic absorption spectrophotometer was used to analyze Na^+^ and K^+^ concentration [8]. Appropriate standards were prepared to maintain the accuracy of the results. The concentration of the standards was 0, 2, 4, 6, 8 and 10 ppm. The Na and K ion concentration was calculated by using the formula:

Na or K = [C ∗ (d ∗ V/1000)]/dwt]

where,

Na or K = Concentration of sodium and potassium ion (mmol/g dwt).
C = Concentration of sample aliquot based on atomic absorption spectrophotometer reading as determined relative to standard curve.
d = dilution factor.
V = extraction volume (mL).
dwt = oven dry weight of the plant leaf (g).

### 4.4. Salinity Scoring and Agronomic Parameters

Salinity scoring was performed to identify the tolerant and sensitive plants based on visual symptoms just after the harvest by using the Standard Evaluation System (SES) [36]. The data on plant height, number of productive and unproductive tillers, panicle length, number of filled and unfilled spikelets and grain yield per plant was determined manually.

### 4.5. Genotyping of Parents and BC_1_F_2_ Progenies

#### 4.5.1. DNA Extraction and Molecular Characterization

Out of 435 BC_1_F_2_ individuals, 190 individuals, not including the tolerant and sensitive parents, were chosen for selective genotyping (choosing the most tolerant and sensitive individuals) from the mapping population based on the visual salinity score (SES) and grain yield per plant under salt stress. From each plant, 25 days old leaves were collected and lyophilized for long time storage [8]. Then, genomic DNA was extracted from those leaf samples using a standard CTAB method [37]. Afterward, DNA quality was checked by agarose gel electrophoresis and quantity was measured by a Nanodrop spectrophotometer. 

Genotyping by skim sequencing was performed using the “AgSeq” platform at the Genomics and Bioinformatics Services at Texas A&M AgriLife Research. Genotyping by skim sequencing employs next-generation sequencing to obtain single nucleotide polymorphism (SNP) marker data on the population [38]. Using this approach, genotype maps for the entire genomes can be developed, which makes it possible to detect that which part of the genome was inherited from each of the parental individuals. In total, 190 BC_1_F_2_ individuals and the two parents were used for skim-based resequencing, and PR106::IRGC53418-1 was used for the genome sequence alignment as it has a high-quality genome assembly for the *indica* rice subgroup [39].

#### 4.5.2. Linkage Map Construction 

The high-density linkage maps of 12 chromosomes were created based on genotypic data of 190 BC_1_F_2_ individuals from the cross between Hasawi and BRRI dhan28, with 6209 polymorphic SNP markers using IciMapping software version 4.2 (https://www.isbreeding.net/software/?type=detail&id=28, accessed on 10 February 2020) based on recombination frequency. Initially, millions of markers per chromosome were filtered using TASSEL (Traits Analysis by Association, Evolution and Linkage) software (https://www.maizegenetics.net/tassel, accessed on 10 February 2020). Firstly, the VCF file for each chromosome was uploaded into TASSEL and the following criteria was selected for filtering the unwanted markers: minimum count was 192; minimum allele frequency was 0.05, and maximum allele frequency was 1.0; minimum and maximum heterozygous proportion were 0.05 and 1.0, respectively. The SNP ordering method used the ordering algorithm of RECORD as previously proposed [40]. After SNP ordering, rippling was done with the COUNT algorithm for fine-tuning of the linkage map. Total length of the distribution of the 6209 markers was 1556.57 cM or 389.14 Mb.

#### 4.5.3. QTL Mapping for Salinity Tolerance

QTL mapping was done using IciMapping software version 4.2 (https://www.isbreeding.net/software/?type=detail&id=28, accessed on 10 February 2020). To identify putative QTLs for salinity tolerance, inclusive composite interval mapping (ICIM-ADD) was used to determine the association between individual marker loci and QTLs. A LOD threshold score of 3.0 and interval map distances based on the result of linkage map analysis were used to determine the association between markers and QTL. In addition, a critical threshold value for QTL detection was calculated by 1000 random permutations of the phenotypic data to establish an experiment-wise significance value at 0.05 [41], i.e., the data were permuted 1000 times to confirm the presence of each QTL across the 12 chromosomes. The percentage of total phenotypic variation explained by QTL identified for each trait was estimated as R^2^ value, and additive effects were also determined for each trait. The QTLs were named using the procedure suggested by McCouch et al. (1997) and McCouch and CGSNL (2008) [42,43].

### 4.6. Candidate Gene Analysis

Q-TARO database (http://qtaro.rd.naro.go.jp/ogro, accessed on 10 February 2020) was used for the identification of candidate genes of nearby quantitative trait locus (QTLs) identified in this study. The compiled gene information table was used for direct comparisons of the identified QTLs. In addition, QTL Genome Viewer was used to view the genomic location of identified QTLs as well as nearby candidate genes or QTLs within a 200 kilobase (kb) region.

### 4.7. Statistical Analysis

Basic descriptive statistics, including mean, standard error, range, and skewness, were performed with Microsoft Excel 2016 and JMP software. Pearson’s correlation analysis for agronomic traits of the BC_1_F_2_ population was performed with R software using the salinity treated genotypes. The mean values of different agronomic parameters under control and salinity treatments were compared with Tukey–Kramer HSD test.

## 5. Conclusions

A total of 40 QTLs related to agronomic and physiological components under salinity stress at the reproductive stage were identified for all traits through inclusive composite interval mapping (ICIM) using the Hasawi × BRRI dhan28 population. Most of the QTLs identified for productive tillers, number of filled spikelets and grain yield were located on chromosomes 3 and 6. In total, eight QTLs for grain yield, seven QTLs for number of filled spikelets, six QTLs for productive tillers, three QTLs each for plant height and percent filled spikelets, twelve QTLs for Na^+^-K^+^ ratio and a solo QTL for the number of unfilled spikelets were identified. Both the tolerant (Hasawi) and sensitive (BRRI dhan28) parents contributed alleles to the QTLs for all traits except the plant height, suggesting that beneficial alleles can be found in both parents. Out of 40 QTLs in Hasawi × BRRI dhan28; two each for plant height, and percent filled spikelets, and three QTLs for productive tillers were statistically confirmed using a stringent 1000 permutation threshold. Overall, the QTLs identified in this study for reproductive-stage salt tolerance show promise for use in marker-assisted selection in future breeding programs to increase selection efficiency. Moreover, identifying the genes underlying these major QTLs would help to better understand the molecular mechanisms controlling reproductive stage salinity tolerance in rice.

## Figures and Tables

**Table 1:** QTLs identified in a Hasawi × BRRI dhan28 population using Inclusive Composite Interval Mapping (ICIMapping) with a LOD 3.0 threshold for salt stress at the reproductive stage of rice.

Trait	Chr.	cM	QTL ^1^	Flanking Markers	LOD	PVE (%)	Additive Effect	Allele Effect
Plant height	**1**	**138.7**	***qPH1.1***	**CM020682.1_33842918_1; CM020682.1_34965920_1**	**14.9**	**27.0**	**−22.46**	**Hasawi**
	**2**	**91.2**	***qPH2.1***	**CM020683.1_22803914_2; CM020683.1_23139808_2**	**7.5**	**9.6**	**−12.36**	**Hasawi**
	4	34.4	*qPH4.1*	CM020685.1_6844517_4; CM020685.1_8787440_4	3.1	3.9	−9.00	Hasawi
Productive tillers	**2**	**7.2**	***qPT2.1***	**CM020683.1_1744356_2; CM020683.1_2059119_2**	**7.0**	**11.2**	**−0.01**	**Hasawi**
	**3**	**27.1**	***qPT3.1***	**CM020684.1_6731611_3; CM020684.1_6810365_3**	**6.1**	**11.9**	**−0.34**	**Hasawi**
	6	118.9	*qPT6.1*	CM020687.1_29137956_6; CM020687.1_29925158_6	4.8	11.7	3.79	BRRI dhan28
	8	31.2	*qPT8.1*	CM020689.1_7802407_8′ CM020689.1_8320104_8	4.2	5.6	4.12	BRRI dhan28
	**11**	**26.4**	***qPT11.1***	**CM020692.1_6481151_11; CM020692.1_6638823_11**	**7.2**	**13.4**	**3.23**	**BRRI dhan28**
	11	20.4	*qPT11.2*	CM020692.1_4890980_11; CM020692.1_5380140_11	3.6	8.6	0.37	BRRI dhan28
No. filled spikelets	**3**	**27.1**	***qNFS3.1***	**CM020684.1_6731611_3; CM020684.1_6810365_3**	**6.6**	**17.9**	**−6.26**	**Hasawi**
	3	87.1	*qNFS3.2*	CM020684.1_21729110_3; CM020684.1_21917452_3	3.9	8.2	−241.51	Hasawi
	6	97.9	*qNFS6.1*	CM020687.1_24336222_6; CM020687.1_24647518_6	3.6	11.5	12.88	BRRI dhan28
	7	52.8	*qNFS7.1*	CM020688.1_12463596_7; CM020688.1_13718255_7	3.5	17.4	−129.05	Hasawi
	8	4.2	*qNFS8.1*	CM020689.1_949460_8; CM020689.1_1275124_8	3.8	16.6	63.01	BRRI dhan28
	11	76.4	*qNFS11.1*	CM020692.1_18857719_11; CM020692.1_19789359_11	4.0	12.3	−509.21	Hasawi
	12	82.2	*qNFS12.1*	CM020693.1_19382691_12; CM020693.1_21580067_12	3.0	15.0	−435.44	Hasawi
No. unfilled spikelets	2	10.2	*qNUFS2.1*	CM020683.1_2221957_2; CM020683.1_2585844_2	5.3	11.4	−229.78	Hasawi
Percent filled spikelets	**4**	**9.4**	***qPFS4.1***	**CM020685.1_2019282_4; CM020685.1_3262402_4**	**4.3**	**14.3**	**11.78**	**BRRI dhan28**
	4	99.4	*qPFS4.2*	CM020685.1_24786787_4; CM020685.1_25615450_4	3.6	6.5	4.60	BRRI dhan28
	**6**	**111.9**	***qPFS6.1***	**CM020687.1_26815088_6; CM020687.1_28217292_6**	**7.8**	**14.9**	**7.55**	**BRRI dhan28**
Grain yield	1	123.7	*qGY1.1*	CM020682.1_30842423_1; CM020682.1_31267467_1	6.2	7.8	−0.44	Hasawi
	**3**	**27.1**	***qGY3.1***	**CM020684.1_6731611_3; CM020684.1_6810365_3**	**7.3**	**11.6**	**−0.10**	**Hasawi**
	3	87.1	*qGY3.2*	CM020684.1_21729110_3; CM020684.1_21917452_3	4.5	5.8	−4.33	Hasawi
Grain yield	3	2.1	*qGY3.3*	CM020684.1_510306_3; CM020684.1_528354_3	3.6	4.3	2.62	BRRI dhan28
	4	98.4	*qGY4.1*	CM020685.1_24368860_4; CM020685.1_24786787_4	4.3	10.0	−1.16	Hasawi
	6	42.9	*qGY6.1*	CM020687.1_10644021_6; CM020687.1_10866598_6	7.1	19.2	0.32	BRRI dhan28
	6	106.9	*qGY6.2*	CM020687.1_24647518_6; CM020687.1_26815006_6	4.5	5.0	2.09	BRRI dhan28
	9	75.1	*qGY9.1*	CM020690.1_18345383_9; CM020690.1_19048905_9	4.1	4.7	2.20	BRRI dhan28
Na-K ratio	1	163.7	*qNaK1.1*	CM020682.1_40556648_1; CM020682.1_41115782_1	12.6	1.9	−3.16	Hasawi
	1	171.7	*qNaK1.2*	CM020682.1_42531739_1; CM020682.1_43167854_1	11.4	1.8	0.28	BRRI dhan28
	3	105.1	*qNaK3.1*	CM020684.1_25664106_3; CM020684.1_27320499_3	11.0	1.9	0.34	BRRI dhan28
	3	119.1	*qNaK3.2*	CM020684.1_29467527_3; CM020684.1_30649884_3	28.8	3.0	0.23	BRRI dhan28
	4	105.4	*qNaK4.1*	CM020685.1_26212611_4; CM020685.1_26658501_4	10.9	1.9	0.00	BRRI dhan28
	4	141.4	*qNaK4.2*	CM020685.1_34446513_4; CM020685.1_36125683_4	12.9	1.8	0.13	BRRI dhan28
	5	79.7	*qNaK5.1*	CM020686.1_18974331_5; CM020686.1_20934712_5	13.6	1.8	−3.13	Hasawi
	6	118.9	*qNaK6.1*	CM020687.1_29137956_6; CM020687.1_29925158_6	11.0	1.9	−0.27	Hasawi
	7	110.8	*qNaK7.1*	CM020688.1_27366179_7; CM020688.1_28119385_7	10.7	1.9	−0.18	Hasawi
	9	3.1	*qNaK9.1*	CM020690.1_371439_9; CM020690.1_1222229_9	12.1	1.7	−3.32	Hasawi
	11	56.4	*qNaK11.1*	CM020692.1_13310544_11; CM020692.1_14431043_11	11.6	1.9	−3.03	Hasawi
	12	7.2	*qNaK12.1*	CM020693.1_1735537_12; CM020693.1_1862734_12	9.2	1.7	−3.39	Hasawi

^1^ Note: QTLs in bold font also passed the 1000 permutation threshold.

## Data Availability

The data presented in this study are available within the published article and the Supplementary Materials.

## Supplementary Materials

The following are available online at https://www.mdpi.com/article/10.3390/ijms231911376/s1.
