# Supplementary material for: Mapping QTLs for Reproductive Stage Salinity Tolerance in Rice Using a Cross between Hasawi and BRRI dhan28
Source: Int J Mol Sci. 2022 Sep 27;23(19):11376. doi: 10.3390/ijms231911376 (PMC9569796; doi:10.3390/ijms231911376)
Supplement: Supplementary file 1 [file ijms-23-11376-s001.zip › ijms-1920866-supplementary.pdf]

Supplementary Materials

Supplementary Table S1. Descriptive Statistics of the BC_1F_2 Progenies of Hasawi x BRRI dhan28 under No-stress and Salinity Stress at Reproductive Stage of Rice.

Traits	Parents		BC_1F_2 Progenies of Hasawi x BRRI dhan28							Decrease
	Hasawi	BRRI dhan28	Mean	Range	Skewness	SE	No-Stress	Tolerant	Sensitive	Sensitive over Tolerant (%)
Plant Height (cm)	124	90	102.02	37-180	0.36	1.61	-	116.31	98.27	15.51
Productive Tillers/plant	18	25	12.65	1 - 39	0.44	0.33	-	17.53	10.49	40.14
Filled Spikelets/plant	1708	2349	307.59	0-2115	1.89	16.54	637.48	735.72	123.59	83.20
Unfilled Spikelets/plant	436	617	633.4	17-3316	1.40	25.39	448.87	793.55	592.84	25.29
Filled Spikelets (%)	79.66	79.2	29.36	0-84.7	0.42	0.95	59.14	49.69	20.64	58.46
Grain Yield (g/plant)	47.97	54.98	5.85	0-43.49	2.11	0.34	16.48	15.06	2.13	85.85
Na^+-K^+ Ratio	1.927	3.105	0.86	0.03-14.5	4.46	0.08	-	0.52	1.18	-128.56

Note: Mean, range, skewness, and standard error (SE) are based on 435 BC_1F_2 individuals grown under salinity stress at the reproductive stage; averages are also shown for a set of 153 BC_1F_2 individuals grown under non-stress control conditions ("no-stress"), along with subsets of BC_1F_2 individuals grown under salinity stress categorized as "tolerant" or "sensitive" by SES scores and grain weight per plant.

Supplementary Table S2. Comparison of Agronomic and Physiological Parameters of the BC1F2 Genotypes of Hasawi x BRRI dhan28 under No-stress and Salinity Stress at Reproductive Stage of Rice (values with the same letter in a column are not significantly different at 5% level of significance).

Treatment	BC_1F_2 Genotypes/ Progenies	Plant Height (cm)	Productive Tillers (no/plant)	Filled Spikelets (no/plant)	Unfilled Spikelets (no/plant)	Filled Spikelets (%)	Unfilled Spikelets (%)	Grain Yield (g/plant)	Na-K Ratio
**Selected Genotypes**									
No-Stress	Control	-	-	637.5^a	448.9^c	59.1^a	40.9^c	16.5^a	-
Salinity- Stress	Tolerant	116.3^a	17.5^a	735.7^a	793.6^a	49.7^b	50.3^b	15.1^a	0.52^b
	Sensitive	98.3^b	10.5^b	123.6^b	592.9^b	20.6^c	79.4^a	2.1^b	1.18^a
$HSD_{0.05}$	Tolerant vs Sensitive	9.52	1.79	125.19	159.79	5.65	5.65	3.32	0.55
	Control vs Tolerant			118.10	151.65	5.33	5.33	3.13	
	Control vs Sensitive			105.56	135.75	4.77	4.77	2.80	
**All Genotypes**									
No-Stress (N)		-	-	637.5^A	448.9^A	59.1^A	40.9^B	16.5^A	-
Salinity Stress (S)		102	12.7	307.6^B	633.4^B	29.4^B	70.6^A	5.9^B	-
$HSD_{0.05}$				69.90	90.74	3.51	3.51	1.66	

Note: For All Genotypes, 153 progenies of BC1F2 population were grown under no-stress/control condition and 435 progenies were grown under salinity stress of EC 10 dS/m; and for Selective Genotypes, number of tolerant and sensitives progenies were 78 and 112, respectively grown under salinity stress of EC 10 dS/m at reproductive stage of rice.

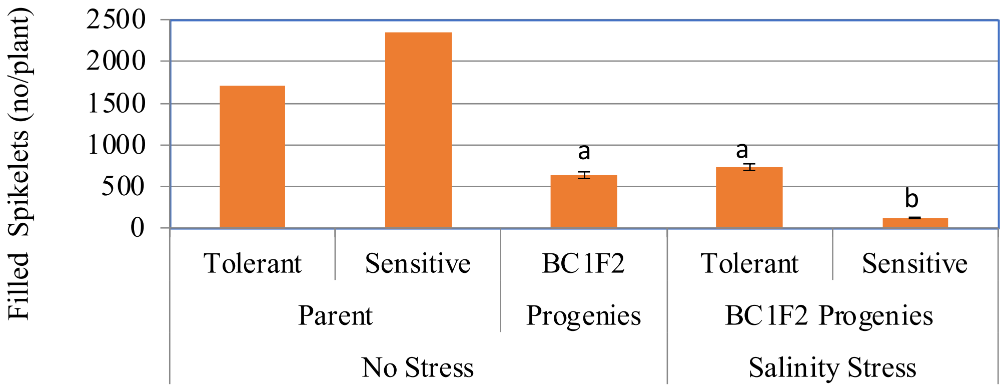

Supplementary Figure S1. Number of filled spikelets per plant of tolerant and sensitive parents under non-stress conditions and their BC_1F_2 progenies under non-stress and salt stress conditions. Vertical and capped bars indicate standard error of the mean number of filled spikelets of 153 plants under no-stress, and 78 tolerant and 112 sensitive progenies grown under salinity stress. Values with the same letter are not significantly different at 5% level of significance.

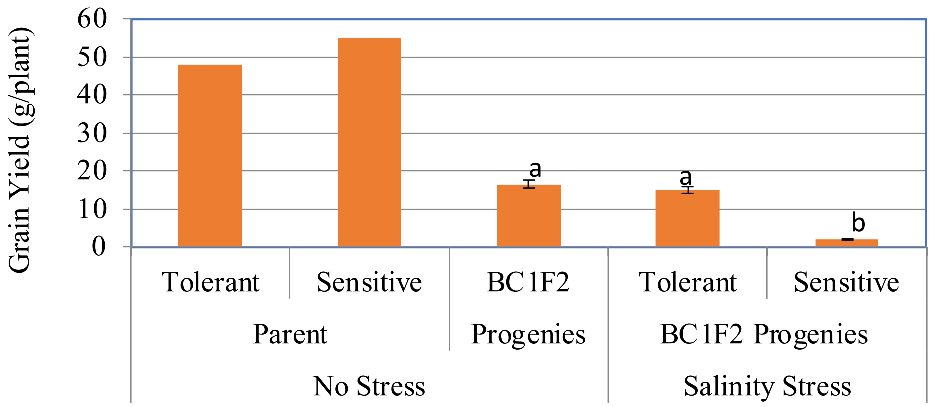

Supplementary Figure S2. Grain yield of tolerant and sensitive parents under non-stress conditions and their BC_1F_2 progenies of Hasawi x BRRI dhan28 under non-stress and salinity salt stress conditions. Vertical and capped bars indicate standard error of the mean grain yield of 153 progenies under no stress, and 78 tolerant and 112 sensitive progenies grown under salinity stress. Values with the same letter are not significantly different at 5% level of significance.
